# State-of-the-Art in Integrated Breast Imaging

**DOI:** 10.1155/2019/7596059

**Published:** 2019-01-06

**Authors:** Graziella Di Grezia, Gianluca Gatta, Luca Brunese, Giuseppe Falco

**Affiliations:** ^1^Radiology Department, “G. Criscuoli” Hospital, Sant'Angelo dei Lombardi, Italy; ^2^Radiology Department, “University of Campania Luigi Vanvitelli”, Naples, Italy; ^3^Medicine and Health Science Department, “University of Molise”, Campobasso, Italy; ^4^Breast Surgery Department, Arcispedale Santa Maria Nuova, Reggio Emilia, Italy

Innovation in radiology is a constant, thanks to technology evolution. Spread research and the daily request of the marketplace and of the other specializations necessitate always new possibilities not only in diagnostic but also in therapeutic fields [1].

Breast cancer represents the first oncological disease for the women and despite the efforts to promote early diagnosis, also with screening programs, advanced disease is already diagnosed.

Estrogen/Progestinic therapies, aesthetic surgery, and the greater life expectancy must be followed by a multimodality approach with a clinical exam and an advanced technology set of exams.

A personalized approach is gradually replacing the “one size fits all” of the previous prevention programs [2]; a breast radiology should answer not only on the diagnosis, but also on follow-up, response to therapy prediction, and therapeutic procedure as alternative to surgery [3].

In this issue we deal with 2D and tomosynthesis evolution, to US/MR coregistration and to features for radiomic approach and laser therapy in nonsurgical old patients.

In detail, Q. Ling et al. in the study* “*Patch Based Grid Artifact Suppressing in Digital Mammography*”* present a solution for fast suppressing grid artifacts and consequently high quality digital mammography.

This is a valid possibility of improving imaging quality also in hospitals and clinics that do not have tomosynthesis or in screening programs.

To date, in some countries there is already a dispute on dose problem in tomosynthesis exams; however, the increase is really minimal and some authors (T. Gomi and Y. Koibuchi in* “*Use of a Total Variation Minimization Iterative Reconstruction Algorithm to Evaluate Reduced Projections during Digital Breast Tomosynthesis*”*) have evaluated the efficacies of the iterative reconstruction algorithm that allows reducing number of projections and reduce radiation doses [4].

Mammography is only the first step, especially in over forty women and in screening programs; however more studies have been conducted to optimize breast MRI results that is to date a very sensible but low specific exam.

R. Fusco et al. in the manuscript* “*Use of Quantitative Morphological and Functional Features for Assessment of Axillary Lymph Node in Breast Dynamic Contrast-Enhanced Magnetic Resonance Imaging*”* evaluated morphologic features and dynamic behavior to predict metastatic disease with a good diagnostic accuracy.

One of the long-standing problems is the US second look to identify additional breast lesions detected on MRI.

The different position of the patient, breast size, and the small size of some lesions on MRI do not allow concluding the diagnosis on second look and in some cases, a MRI guided biopsy could be necessary.

A. Mazzei et al. in the study “Efficacy of Second-Look Ultrasound with MR Coregistration for Evaluating Additional Enhancing Lesions of the Breast: Review of the Literature” present that the coregistration of US and MRI allows reducing these problems also thanks to multiplanar reconstructions.

Therapeutic possibilities have been addressed in the study* “*The Evolving Role of Ultrasound Guided Percutaneous Laser Ablation in Elderly Unresectable Breast Cancer Patients: A Feasibility Pilot Study” by J. Nori et al.

Interventional procedures are not always and only diagnostic [5]; laser ablation is a really alternative to surgery in old patients with high anesthesiological risk and not eligible to surgery with a good compliance of the patients, less complications, and shorter hospitalization in comorbidity patients.

In the future the radiologist should predict the therapy response to breast cancer and orient geneticists [6, 7], oncologist, radiotherapist, and surgeons on the best personalized approach to breast cancer in specific patients. It would be possible thanks to radiomics features and multidisciplinary approach including biomedical engineers and physics (P. Crivelli et al. in “A New Challenge for Radiologists: Radiomics in Breast Cancer”) [8].
